# Predictors of resistance and mortality in *Klebsiella pneumoniae* infections: a matched case–case–control study

**DOI:** 10.3389/fmed.2026.1828578

**Published:** 2026-07-17

**Authors:** Hadi Al Sulayyim, Mohammed Bazuqamah, Seham Eldeeb, Sherif Khorshid, Abdullah Al Hamid

**Affiliations:** 1 Infection Prevention and Control Department, Najran Health Cluster, Najran, Saudi Arabia; 2 Microbiology Department, King Khalid Hospital, Najran, Saudi Arabia; 3 Public Health and Community Medicine Department, Zagazig University, Zagazig, Egypt; 4 Infection Prevention and Control Department, King Khalid Hospital, Najran, Saudi Arabia; 5 College of Clinical Pharmacy, King Faisal University, Alahsa, Saudi Arabia

**Keywords:** Carbapenem resistance, case–case–control, *Klebsiella pneumoniae*, mortality, risk factors

## Abstract

**Background:**

Carbapenem-resistant *Klebsiella pneumoniae* (CRKP) is a major public-health threat associated with limited treatment options and increased mortality. This study identified risk factors for CRKP positive urine cultures, compared with carbapenem-susceptible *K. pneumoniae* positive urine cultures (CSKP), and assessed predictors of death.

**Methods:**

A retrospective matched case–case–control study was conducted at a referral hospital in Najran, Saudi Arabia, between 1 January–31 December 2024. Adults with positive urine cultures of CRKP and CSKP were compared with matched controls without *K. pneumoniae* infection. Matching used hospital location/department, 30-day window period, specimen type, and time at risk; controls were additionally matched on age. Exploratory multivariable logistic regression identified adjusted associations.

**Results:**

In exploratory adjusted models, CRKP positive urine culture episodes showed association with prolonged hospital stay/hospital settings (AOR 1.015/6.599), and several comorbidities: pulmonary disease (AOR 5.952), renal disease (AOR 3.355), and malignancy (AOR 13.420). The malignancy estimate in the CRKP vs. control model was imprecise and was treated as an exploratory signal rather than a stable adjusted predictor. Demographic predictors for CRKP included female sex (AOR 6.172) and non-Saudi nationality (AOR 7.160). Similarly, CSKP positive urine culture episodes were associated with female sex, non-Saudi nationality, renal disease; malignancy, hepatic disease (AOR 8.100) and absence of device use (AOR 9.330) produced imprecise estimates and were interpreted only as exploratory signals. When comparing CRKP to CSKP, carbapenem resistance was associated with older age (AOR 1.021), diabetes mellitus (AOR 2.689); hospital setting (AOR 36.955) estimate suggested a healthcare-associated direction but had a wide confidence interval and unstable magnitude. Mortality was higher in CRKP (18.1%) than CSKP (9.2%) and controls (9.7%), but this did not reach statistical significance in the unadjusted group comparison (*p* = 0.062). The mortality model showed associations with advancing age (OR 1.038 per year), hospital setting (OR 3.852), carbapenem resistance (OR 2.844), cardiovascular disease (OR 3.302), and device utilization (OR 5.522).

**Conclusion:**

CRKP was predominantly healthcare-associated and more likely among older patients and those with co-comorbidities. Since important confounders were unavailable and several estimates were imprecise, these findings should be interpreted as exploratory and hypothesis-generating.

## Introduction

*Klebsiella pneumoniae* (*K. pneumoniae*) is one of the normal commensal organisms, which live on human mucosal surfaces, including the oropharynx and gastrointestinal tract. It can invade other tissues and result in serious infections such as bloodstream infection, wound or surgical site infection, pneumonia, urinary tract infection, and meningitis (1).

Carbapenems are a class of antimicrobial agents that includes imipenem, meropenem, and ertapenem. They are used as a first-line choice to treat severe infections due to *K. pneumoniae*. However, carbapenem-resistant *K. pneumoniae* (CRKP) has now become a serious threat to human health. CRKP is currently a considerable worldwide public health concern that is associated with increased rates of morbidity and mortality (2). Moreover, failure of therapeutic options, extended hospital length of stay, and high medical expenses are considered a burden associated with CRKP (3, 4).

According to a global systematic review and meta-analysis, the prevalence of CRKP among infected patients with *K. pneumoniae* was 28.69%. The highest prevalent rates were in South Asia, whereas the lowest were in high-income North America (5).

In the Kingdom of Saudi Arabia (KSA), the first nosocomial outbreak caused by CRKP was reported in 2012 (6). In Jeddah, KSA, the prevalence of carbapenem-resistant (CRE) in a tertiary hospital was 8% in 2017 and raised to 13% in 2018. The study also mentioned that the prevalence at another hospital was 57% in 2018 and increased to 61% in 2019 (7–9). Local evidence underscores the role of both clinical interventions and pharmacological history in CRE acquisition. Identified risks include CVC placement and surgery (7), alongside recent exposure to carbapenems, quinolones, and piperacillin-tazobactam (7, 8).

While most previous studies have addressed the associated risk factors with healthcare-associated CRE, a few studies were concerned with CRKP. However, most of those studies compared CRKP as cases to carbapenem-sensitive *K. pneumoniae* (CSKP), and there is scarcity of case–control studies in the KSA addressing healthcare CRKP, as well as no study in Najran region studied the risk factors associated with CRKP. Therefore, to minimize biases associated with direct comparison of such infected cases, this study aimed adapting case–case–control design to identify the risk factors associated with CRKP at King Khalid Hospital (KKH); to determine the mortality rate and associated factors.

## Materials and methods

### Study design and population

This study was carried out at a referral hospital located in Najran region, KSA. The hospital has a 450-bed capacity and admits roughly 13,855 patients annually. Najran is a region located in the south-west of the KSA and has approximately 592,300 people.

This study employed a case–case–control design to overcome limitations of studies identifying risk factors for antibiotic resistance (AR) (8). The study was conducted from 1st January 2024 through 31st December 2024 and included all adult patients with CRKP or CSKP positive urine cultures identified during the study period. The design comprised two separate matched case–control comparisons: CRKP cases versus controls without *K. pneumoniae* infection, and CSKP cases versus controls without *K. pneumoniae* infection. CRKP and CSKP cases were not matched 1:1 to each other.

The analysis consisted of two parts: (A) separate matched case–control analyses identifying factors associated with CRKP and CSKP compared with their respective control groups; and (B) case–case and mortality analyses comparing outcomes and characteristics among patients with CRKP and CSKP. The CRKP vs. CSKP comparison was analyzed separately and was not based on the matched strata used for the case–control comparisons.

### Inclusion and exclusion criteria

All adult patients, men and women, positive for CRKP/CSKP with a clinical urine culture yielding CRKP or CSKP during the study period and admitted to or visited the hospital were included. Microbiological cultures that were performed for patients who visited the emergency department and outpatients, as well as those hospitalized, with cultures isolating bacteria other than CRKP and CSKP were excluded.

### Case definition

CRKP was defined as *K. pneumoniae* isolates exhibiting non-susceptibility (intermediate or resistant) to at least one carbapenem (imipenem, meropenem, or ertapenem) according to CLSI M100 breakpoints. CSKP was defined as *K. pneumoniae* isolates susceptible to carbapenems. Although this definition includes intermediate isolates, all CRKP isolates included in the present study were resistant to at least one carbapenem; therefore, application of a resistant-only definition would not have altered case classification. For controls, patients who visited the hospital for any reason within a 30-day window period of CRKP/CSKP cases and did not fulfill the case definition were included.

### Data collection

Although urinary isolates represent a substantial proportion of clinical *K. pneumoniae* isolates encountered in routine practice, this study focused exclusively on urine specimens collected during the study period. No blood, respiratory, wound, screening, or surveillance specimens were included. Controls were selected separately for each case group. For each CRKP case, two controls without *K. pneumoniae*-positive cultures were selected; likewise, for each CSKP case, two controls without *K. pneumoniae*-positive cultures were selected. Controls had hospital contact within the +/−30-day matching window and did not have a clinical culture yielding *K. pneumoniae* during that window. Matching variables were hospital location/department, specimen type, calendar time window of +/−30 days, and time at risk, defined as the interval between hospital admission or encounter and the date of culture collection. Controls were also age-matched as closely as available records allowed. Each matched case–control set was assigned a stratum identifier, and conditional logistic regression incorporated the matching by conditioning on these matched strata. The CRKP-versus-CSKP comparison and mortality analysis were performed using multivariable logistic regression because these analyses were not based on matched case–control sets. Only the first episode was examined for outcome and epidemiological analyses among patients from whom several strains of CRKP/CSKP were isolated over the study period. Only clinical cultures obtained for diagnostic purposes were included. Screening or surveillance cultures were excluded where identifiable. Infection was inferred from recovery of *K. pneumoniae* from a clinical urine specimen obtained during routine clinical care. However, retrospective adjudication using standardized urinary tract infection definitions was not feasible; therefore, differentiation between true infection, asymptomatic bacteriuria, and colonization could not be confirmed for all patients.

Demographic and clinical data included the following variables: (a) demographic data including age, gender, and nationality, (b) clinical data including hospital length of stay (LOS), hypertension, diabetes mellitus, pulmonary disease, renal disease, hepatic disease, cardiovascular disease, central nervous system disease, malignancy, devices being used, and type of bacteria (CRKP/CSKP). Regarding the LOS, it was defined as the total duration (in days) from hospital admission to hospital discharge. This variable reflects the total inpatient exposure period for each patient included in the analytical cohort. For patients with multiple admissions during the study period. LOS was calculated for the admission associated with the index urine culture episode included in the analysis.

### Microbiological identification and antimicrobial susceptibility testing (AST)

*Klebsiella pneumoniae* isolates were recovered from routine clinical urine specimens and identified using standard laboratory procedures, including colony morphology, Gram staining, and automated identification systems (BD Phoenix^™^, Vitek® 2, and MicroScan WalkAway®). Antimicrobial susceptibility testing (AST) was performed using the same automated systems, and the results were interpreted according to the Clinical and Laboratory Standards Institute (CLSI) M100 guidelines. Carbapenem-resistant *K. pneumoniae* (CRKP) was phenotypically defined as any isolate demonstrating intermediate susceptibility or resistance to at least one carbapenem (ertapenem, meropenem, or imipenem). Quality control was maintained throughout the study using standard reference strains, including *Escherichia coli* ATCC 25922, in accordance with established laboratory protocols.

### Carbapenemase gene testing

Molecular testing for carbapenemase genes (OXA-48-like, NDM, VIM, and KPC) was performed using the Cepheid GeneXpert® system on a subset of CRKP isolates during routine clinical work-up. Gene results were reported as single-gene or combined-gene detection patterns. Carbapenemase testing was performed only for a subset of isolates and was not used for case classification. Because carbapenemase gene testing was not performed for all CRKP isolates, phenotypic carbapenem non-susceptibility remained the primary case definition for CRKP in the main analysis.

### Quality control measures

Standard ATCC strains (e.g., *Escherichia coli* ATCC 25922, *Staphylococcus aureus* ATCC 29213) were used for quality control during identification and AST. Media sterility and system calibration were verified prior to testing.

### Bacterial isolates

Clinical isolates of *Klebsiella pneumoniae* identified as CRE were obtained from clinical specimens from hospitalized patients. The isolates confirmed as *K. pneumoniae* based on standard biochemical tests and automated identification systems.

### Mortality modeling

Mortality was evaluated using multivariable logistic regression. Predictors included demographic variables, culture category (CRKP, CSKP, or control), hospital setting, comorbidities, and device use. Given the limited number of events and the absence of specific severity-of-illness scores, this model is interpreted as exploratory.

### Statistics analysis

Categorical variables were summarized as *n* (%) and compared using chi-square or Fisher’s exact tests, and continuous variables as median (Q1–Q3) and compared using Mann–Whitney *U* or Kruskal–Wallis tests, as appropriate. For the matched case–control comparisons, conditional logistic regression was used with the matched set identifier as the stratum variable. Variables with *p* < 0.10 in univariable models (or with strong clinical relevance) were entered into multivariable models. Because age matching was not exact and residual age imbalance remained, age was retained as a covariate in the regression models. Adjusted odds ratios (AORs) with 95% confidence intervals (CIs) are reported. Analyses were conducted using IBM SPSS Statistics (version 28). A two-sided *p* < 0.05 was considered statistically significant. Because of the modest sample size and the limited number of outcome events in some categories, the multivariable models were considered exploratory. CIs or sparse cell counts were not interpreted as stable independent predictors; their results were described as exploratory model signals and interpreted according to direction and precision rather than point estimates alone.

### Ethical considerations

Ethical approval was obtained from the Institutional Review Board of King Khalid Hospital, Najran, Saudi Arabia, before conducting the study (IRB Log No. 2024–33.4). This retrospective study involved analysis of routinely collected clinical and microbiological data. No identifiable patient information was included in the manuscript. The requirement for informed consent was waived by the IRB because of the retrospective nature of the study.

## Results

Demographic, clinical, and outcome characteristics are shown in Table 1. A total of 510 patients were included: 94 CRKP, 76 CSKP, and 340 controls. All CRKP and CSKP isolates included in the analytical cohort originated from urine specimens. Therefore, no blood, respiratory, wound, or other specimen types were represented in the study cohort. Median age did not differ significantly among groups (*p* = 0.732). Median hospital LOS differed significantly among groups (*p* < 0.001), with CRKP patients having the longest stay (median 1 day [Q1–Q3: 0–32]) compared with CSKP patients (median 1 day [0–4]) and controls (median 0 days [0–6]). LOS demonstrated a highly skewed distribution with wide upper quartiles, indicating substantial variability in hospitalization duration despite the low median values. Sex and nationality distributions differed across groups (*p* < 0.001 for both). Across groups, hypertension (*p* = 0.026), pulmonary disease (*p* = 0.002), renal disease (*p* = 0.001), and malignancy (*p* < 0.001) differed significantly. Mortality was higher in CRKP (18.1%) than CSKP (9.2%) and controls (9.7%), but this did not reach statistical significance in the unadjusted group comparison (*p* = 0.062).

**Table 1:** Demographic, clinical, and outcome data of 94 patients with CRKP, 76 patients with CSKP, and 340 control patients.

Parameter	Category	Resistance (*n* = 94)	Sensitive (*n* = 76)	Control (*n* = 340)	
		*N* (%)	*N* (%)	*N* (%)	*p*-value
Age Med (Q1-Q3)		70 (58–78)	66 (25–75)	68 (55–78)	0.732^a^
Length of stay Med (Q1-Q3)		1 (0–32)	1 (0–4)	0 (0–6)	0.000^a,*^
Setting	Com	58 (61.7)	55 (72.4)	222 (65.3)	0.334
	Hosp	36 (38.3)	21 (27.6)	118 (34.7)	
Gender	F	58 (61.7)	50 (65.8)	99 (29.1)	0.000^*^
	M	36 (38.3)	26 (34.2)	241 (70.9)	
Nationality	NS	26 (27.7)	23 (30.3)	29 (8.5)	0.000^*^
	S	68 (72.3)	53 (69.7)	311 (91.5)	
HTN	No	37 (39.4)	29 (38.2)	175 (51.5)	0.026^*^
	Yes	57 (60.6)	47 (61.8)	165 (48.5)	
DM	No	35 (37.2)	35 (46.1)	172 (50.6)	0.069
	Yes	59 (62.8)	41 (53.9)	168 (49.4)	
PD	No	76 (80.9)	69 (90.8)	316 (92.9)	0.002^*^
	Yes	18 (19.1)	7 (9.2)	24 (7.1)	
RD	No	69 (73.4)	57 (75)	298 (87.6)	0.001^*^
	Yes	25 (26.6)	19 (25)	42 (12.4)	
HD	No	88 (93.6)	67 (88.2)	322 (94.7)	0.111
	Yes	6 (6.4)	9 (11.8)	18 (5.3)	
CVD	No	59 (62.8)	52 (68.4)	210 (61.8)	0.554
	Yes	35 (37.2)	24 (31.6)	130 (38.2)	
CNSD	No	72 (76.6)	67 (88.2)	259 (76.2)	0.069
	Yes	22 (23.4)	9 (11.8)	81 (23.8)	
Malig	No	83 (88.3)	68 (89.5)	334 (98.2)	0.000^*^
	Yes	11 (11.7)	8 (10.5)	6 (1.8)	
Devices	No	70 (74.5)	70 (92.1)	265 (77.9)	0.000^*^
Used	Yes	24 (25.5)	6 (7.9)	75 (22.1)	
Outcome	Death	17 (18.1)	7 (9.2)	33 (9.7)	0.062
	Survive	77 (81.9)	69 (90.8)	307 (90.3)	

COM, community; CNSD, central nervous system diseases; CVD, cardiovascular diseases; DM, diabetes mellitus; HD, hepatic disease; Hos, hospital; HTN, hypertension; malig, malignancy; Med, median; NS, non-Saudi; PD, pulmonary disease; RD, renal disease; S, Saudi. a Kruskal wallis; *Significant at *p* = 0.05.

In the exploratory adjusted model, factors associated with CRKP positive urine cultures compared to controls were longer hospital stay (*p* = 0.047), hospital setting (*p* = 0.000), female sex (*p* = 0.000), non-Saudi nationality (*p* = 0.000), pulmonary disease (*p* = 0.004), renal disease (*p* = 0.025), and malignancy (*p* = 0.011). The malignancy estimate was statistically significant but imprecise because of sparse data; it was therefore treated as an exploratory signal only.

In the exploratory multivariable model comparing CRKP (*n* = 94) with matched controls (*n* = 188) (Table 2), adjusted associations with CRKP were: longer LOS (AOR 1.015 per day; 95% CI 1.000–1.029; *p* = 0.047), hospital setting (AOR 6.599; 95% CI 2.711–16.59; *p* < 0.001), female sex (AOR 6.172; 95% CI 3.028–12.581; *p* < 0.001), non-Saudi nationality (AOR 7.160; 95% CI 2.373–21.613; *p* < 0.001), pulmonary disease (AOR 5.952; 95% CI 1.797–19.715; *p* = 0.004), and renal disease (AOR 3.355; 95% CI 1.167–9.650; *p* = 0.025). Malignancy produced an imprecise estimate (AOR 13.420; 95% CI 1.794–100.404; *p* = 0.011), so it was not interpreted as a stable adjusted predictor and is reported as an exploratory model signal only.

**Table 2:** Exploratory multivariate analysis for different clinical and demographic variables for 94 patients with CRKP infections positive urine cultures compared to 188 control patients.

Variable	Category	AOR	(95% CI)	Sig.
Age		0.999	(0.970–1.028)	0.927
Length of stay		1.015	(1.000–1.029)	0.047^*^
Setting	Hos	6.599	(2.711–16.59)	0.000^*^
	Com	1	1	1
Gender	F	6.172	(3.028–12.581)	0.000^*^
	M	Ref	Ref	
Nationality	NS	7.160	(2.373–21.613)	0.000^*^
	S	Ref	Ref	
HTN	Yes	0.604	(0.203–1.792)	0.363
	No	Ref	Ref	
DM	Yes	1.657	(0.630–4.361)	0.306
	No	Ref	Ref	
PD	Yes	5.952	(1.797–19.715)	0.004^*^
	No	Ref	Ref	
RD	Yes	3.355	(1.167–9.650)	0.025^*^
	No	Ref	Ref	
HD	Yes	0.469	(0.092–2.389)	0.362
	No	Ref	Ref	
CVD	Yes	0.735	(0.290–1.862)	0.516
	No	Ref	Ref	
CNSD	Yes	0.726	(0.280–1.882)	0.510
	No	Ref	Ref	
Malig	Yes	13.420	(1.794–100.404)	0.011^*^
	No	Ref	Ref	
Devices used	Yes	2.195	(0.791–6.090)	0.131
	No	Ref	Ref	

COM, community; CNSD, central nervous system diseases; CVD, cardiovascular diseases; DM, diabetes mellitus; HD, hepatic disease; Hos, hospital; HTN, hypertension; malig, malignancy; Med, median; NS, non-Saudi; PD, pulmonary disease; RD, renal disease; S, Saudi. CI, confidence interval; *Significant at *p* = 0.05. Ref, Reference; AOR, Adjusted odds ratio.

In the exploratory comparison of CSKP (*n* = 76) positive urine cultures with matched controls (*n* = 152) (Table 3), adjusted associations with CSKP were: female sex (AOR 7.686; 95% CI 3.482–16.966; *p* < 0.001), non-Saudi nationality (AOR 10.306; 95% CI 3.358–31.632; *p* < 0.001), and renal disease (AOR 3.456; 95% CI 1.161–10.294; *p* = 0.026). The model also produced imprecise estimates for hepatic disease (AOR 8.100; 95% CI 1.485–44.186; *p* = 0.016), malignancy (AOR 9.264; 95% CI 1.624–52.833; *p* = 0.012), and absence of device use (No vs. Yes: AOR 9.330; 95% CI 1.954–44.536; *p* = 0.005); these were not interpreted as stable adjusted predictors and are reported as exploratory signals only.

**Table 3:** Exploratory multivariate analysis for different clinical and demographic variables for 76 patients with CSKP positive urine cultures compared to 152 control patients.

	Category	AOR	(95% CI)	Sig.
Age		0.999	(0.971–1.028)	0.960
Hospital length of stay		1.029	(0.995–1.065)	0.098
Setting	Hos	0.505	(0.171–1.495)	0.217
	Com	Ref	Ref	
Gender	F	7.686	(3.482–16.966)	0.000^*^
	M	Ref	Ref	
Nationality	NS	10.306	(3.358–31.632)	0.000^*^
	S	Ref	Ref	
HTN	Yes	1.765	(0.682–4.569)	0.241
	No	Ref	Ref	
DM	Yes	1.057	(0.454–2.464)	0.897
	No	Ref	Ref	
PD	Yes	1.107	(0.323–3.795)	0.872
	No	Ref	Ref	
RD	Yes	3.456	(1.161–10.294)	0.026^*^
	No	Ref	Ref	
HD	Yes	8.100	(1.485–44.186)	0.016^*^
	No	Ref	Ref	
CVD	Yes	0.935	(0.365–2.395)	0.889
	No	Ref	Ref	
CNSD	Yes	0.500	(0.165–1.513)	0.220
	No	Ref	Ref	
Malig	Yes	9.264	(1.624–52.833)	0.012^*^
	No	Ref	Ref	
Devices used	No	9.330	(1.954–44.536)	0.005^*^
	Yes	Ref	Ref	

COM, community; CNSD, central nervous system diseases; CVD, cardiovascular diseases; DM, diabetes mellitus; HD, hepatic disease; Hos, hospital; HTN, hypertension; malig, malignancy; Med, median; NS, non-Saudi; PD, pulmonary disease; RD, renal disease, S, Saudi. CI, confidence interval; *Significant at *P* = 0.05. Ref, Reference; AOR, Adjusted odds ratio.

In the exploratory case–case model comparing CRKP (*n* = 94) with CSKP (*n* = 76) (Table 4), older age (AOR 1.021 per year; 95% CI 1.004–1.039; *p* = 0.018) and diabetes mellitus (AOR 2.689; 95% CI 1.309–5.521; *p* = 0.007) were associated with CRKP. Hospital setting showed a large positive model signal (AOR 36.955; 95% CI 7.873–173.468; *p* = 0.005), but the very wide CI indicates limited precision and possible sparse-data bias; therefore, it was not interpreted as a stable adjusted predictor, and the direction of association is more reliable than the exact magnitude.

**Table 4:** Exploratory multivariate analysis for different clinical and demographic variables for 94 patients with CRKP infections positive urine cultures compared to 76 patients with CSKP positive urine cultures infections.

	Category	AOR	(95% CI)	Sig.
Age		1.021	(1.004–1.039)	0.018^*^
Hospital length of stay		0.998	(0.994–1.002)	0.415
Setting	Hospital	36.955	(7.873–173.468)	0.005^*^
	Community	Ref	Ref	
Gender	Male	0.593	(0.310–1.136)	0.115
	Female	Ref	Ref	
Nationality	Non-Saudi	1.258	(0.630–2.511)	0.516
	Saudi	Ref	Ref	
Hypertension	Yes	0.661	(0.299–1.462)	0.307
	No	Ref	Ref	
Diabetes mellitus	Yes	2.689	(1.309–5.521)	0.007^*^
	No	Ref	Ref	
Pulmonary disease	Yes	2.054	(0.781–5.405)	0.145
	No	Ref	Ref	
Renal disease	Yes	0.857	(0.405–1.812)	0.686
	No	Ref	Ref	
Hepatic disease	Yes	0.672	(0.245–1.848)	0.442
	No	Ref	Ref	
Cardiovascular disease	Yes	0.700	(0.349–1.404)	0.315
	No	Ref	Ref	
Central nervous system disease	Yes	2.084	(0.957–4.536)	0.064
	No	Ref	Ref	
Malignancy	Yes	0.701	(0.275–1.789)	0.457
	No	Ref	Ref	
Devices being used	Yes	2.343	(0.928–5.914)	0.072
	No	Ref	Ref	

CI, confidence interval; *Significant at *p* = 0.05. Ref, Reference; AOR, Adjusted odds ratio.

In the multivariable model for mortality (Table 5), higher odds of death were associated with older age (OR 1.038 per year; 95% CI 1.008–1.068; *p* = 0.010), hospital setting (OR 3.852; 95% CI 1.820–8.152; *p* < 0.001), carbapenem resistance (CRKP vs. controls: OR 2.844; 95% CI 1.217–6.645; *p* = 0.016), cardiovascular disease (OR 3.302; 95% CI 1.539–7.082; *p* = 0.002), and device use (Yes vs. No: OR 5.522; 95% CI 2.642–11.544; *p* < 0.001).

**Table 5:** Exploratory variables associated with death.

Variable	Category	AOR	95% CI	*p*-value
Age		1.038	(1.008–1.068)	0.010*
Length of stay		1.001	(0.996–1.001)	0.628
Setting	Hos	3.852	(1.820–8.152)	0.000*
	Com	Ref	Ref	
Resistance type	Sensitive	2.156	(0.754–6.164)	0.152
	Resistance	2.844	(1.217–6.645)	0.016*
	No growth	Ref	Ref	
HTN	Yes	1.393	(0.549–3.535)	0.485
	No	Ref	Ref	
DM	Yes	1.320	(0.588–2.962)	0.500
	No	Ref	Ref	
HD	Yes	2.065	(0.743–5.738)	0.164
	No	Ref	Ref	
CVD	Yes	3.302	(1.539–7.082)	0.002*
	No	Ref	Ref	
Devices used	Yes	5.522	(2.642–11.544)	0.000*
	No	Ref	Ref	

COM, community; CVD, cardiovascular diseases; DM, diabetes mellitus; HD, hepatic disease; Hos, hospital; HTN, hypertension; CI, confidence interval; *Significant at *p* = 0.05. Ref, Reference; AOR, Adjusted odds ratio.

In the carbapenemase testing within the analytical Cohort, among the 94 CRKP patients included in the analytical cohort, 53 (56.4%) underwent carbapenemase gene testing as part of routine clinical care, whereas 41 (43.6%) were not tested. Among the tested isolates, OXA-48-like alone was detected in 22 (41.5%), combined NDM and OXA-48-like in 18 (34.0%), NDM alone in 11 (20.8%), triple detection (VIM, NDM, and OXA-48-like) in 1 (1.9%), and combined NDM and KPC in 1 (1.9%).

In the supplementary institutional surveillance data separating from the analytical study cohort, 199 CRKP isolates underwent GeneXpert carbapenemase testing during the study period as part of routine clinical and infection-control surveillance activities. These isolates were not included in the matched case–case–control analyses. The distribution was as follows: OXA-48-like alone, 104 (52.3%); combined NDM and OXA-48-like, 54 (27.1%); NDM alone, 34 (17.1%); combined VIM and OXA-48-like, 3 (1.5%); combined VIM and NDM, 2 (1.0%); combined NDM and KPC, 1 (0.5%); and VIM alone, 1 (0.5%).

## Discussion

In this retrospective case–case–control study conducted at KKH, Najran, we analyzed 94 CRKP cases, 76 CSKP cases, and 340 matched controls. In the exploratory adjusted model comparing CRKP with controls, longer LOS, hospital setting, female sex, non-Saudi nationality, pulmonary disease, and renal disease showed adjusted associations with CRKP positive urine culture cases. Malignancy generated an imprecise model signal and was not interpreted as a stable adjusted predictor. Overall, these results should be interpreted as exploratory associations rather than definitive independent predictors.

Our findings align with much of the global literature on risk factors for CRKP infection, while also highlighting some unique associations. Prolonged hospitalization has consistently emerged as a key risk factor for CRKP acquisition (10). Prior studies in various settings have reported that a longer LOS, especially in intensive care units (ICUs), greatly increases the odds of acquiring CRKP. This is understandable, as extended and critical care stays often entail greater exposure to broad-spectrum antibiotics and invasive devices. In our study, the apparently short median LOS should be interpreted in the context of a highly skewed distribution. Several patients had LOS values of 0–1 day because they were evaluated in the emergency department, discharged on the same day, transferred from other healthcare facilities, admitted for short observation periods, or died shortly after presentation. In contrast, a smaller subgroup experienced prolonged hospitalization, resulting in wide upper quartiles despite low median values. The association with longer hospital stay (even after matching) reinforces the notion that hospital exposure duration is a primary driver of CRKP risk, consistent with systematic reviews and large multicenter analyses (11).

Hospital versus community setting was non-significant in the overall Table 1 comparison (*p* = 0.334), yet it became a strong predictor in multivariable models. This pattern suggests confounding in the crude comparison. However, the very wide CI for the hospital setting in the CRKP-versus-CSKP model also indicates limited precision and possible sparse-data bias. Therefore, this finding supports a healthcare-associated direction of effect, but the exact magnitude should not be interpreted as stable and should be validated in larger multicenter studies.

Similarly, the strong association we found with a “hospital setting” (i.e., healthcare-associated acquisition) is in line with global evidence that most CRKP infections are nosocomial. For example, an international case–control study identified prior admission to a healthcare facility (e.g., transfer from another hospital or long-term care facility) as a major risk factor for CRE/Klebsiella infections (12). These parallels suggest that our hospital’s CRKP cases arose from the same general risk context reported worldwide: prolonged and intensive healthcare exposure.

The multivariate analysis identified several host factors that could associate with the acquisition of CSKP infections. Notably, female sex was a significant risk factor – females had markedly higher odds of CSKP infection compared to males (AOR = 7.7, *p* < 0.001). This finding aligns with some reports that *K. pneumoniae* infection rates can be slightly higher in women (13), potentially due to females’ predisposition to urinary tract infections (UTIs), where Klebsiella can be a pathogen. In contrast, prior population-based studies of Klebsiella bacteremia have often found that male sex is at higher risk (14), suggesting that the female predominance observed in our study may relate to the type of infection (more community-onset UTI cases in women) rather than a generalizable gender effect.

Another notable demographic factor was nationality. Non-Saudi patients were 10 times more likely to acquire CSKP infection than Saudi patients in our cohort (AOR = 10.3, *p* < 0.001). This striking association has not been widely reported elsewhere and likely reflects context-specific dynamics. This association should be interpreted cautiously as nationality is unlikely to be a biological risk factor and may represent unmeasured differences in healthcare access, occupational exposure, living conditions, comorbidity burden, or healthcare-seeking behavior. Because these variables were not available, no causal inference can be made.

An unexpected finding in our analysis was that lack of device use was associated with a greater risk of CSKP infection (*p* = 0.005). Patients who did not have any supportive devices (such as catheters or ventilators) had significantly higher odds of infection compared to those who had used invasive devices (AOR = 9.3). However, the confidence interval was wide, and this finding was not interpreted as a stable adjusted predictor. At first glance, this appears counterintuitive, as indwelling devices are traditionally known to facilitate the growth of Klebsiella and other hospital-acquired infections (13). The association between device nonuse and CSKP infection should be interpreted cautiously. This finding is counterintuitive and may reflect residual confounding, differences between community-onset and hospital-onset infections, or selection bias rather than a true protective effect of device use. Because detailed data on the source of infection, timing of device insertion, illness severity, and prior healthcare exposure were unavailable, no causal interpretation is possible. The observed association between device non-use and CSKP should not be interpreted causally. It may reflect residual confounding, differences between community-onset and healthcare-associated presentations, or other unmeasured clinical factors that were unavailable in the dataset.

Although the present study did not evaluate environmental exposures directly, Previous reports from Yemen have documented challenges related to drinking-water quality, wastewater management, environmental contamination, and antimicrobial use practices, which may contribute to the dissemination of antimicrobial-resistant organisms in the region. However, these factors were not evaluated in the present study and therefore no causal inference can be made. (15, 16).

Other studies have indeed shown that typical risk factors for *K. pneumoniae* infection include invasive hospital interventions (central lines, urinary catheters, ventilation, etc.) (10). It underlines that community-acquired CSKP infections – occurring in patients without prior devices – constitute a significant portion of the burden and should not be overlooked.

Our study also investigated factors that distinguish CRKP infections from those caused by susceptible strains. In the exploratory CRKP vs. CSKP case–case model, older age and diabetes mellitus showed adjusted associations with CRKP. Hospital setting also showed a positive model signal, but the estimate was imprecise because of sparse data and was therefore interpreted directionally rather than as a stable adjusted predictor.

The infection setting generated one of the largest adjusted model signals for resistance. In our data, hospital-setting infections were more likely to be CRKP than community-setting infections; however, the very wide confidence interval around this estimate indicates sparse data and unstable effect-size estimation. This result was therefore interpreted as directional support for a healthcare-associated pattern of CRKP rather than as a stable estimate of magnitude. Hospital environments, particularly intensive care units, combine high antibiotic selective pressure, invasive procedures, and opportunities for cross-transmission. Accordingly, ICU admission has been identified as an independent risk factor for MDR and CRKP infections in prior studies. Our results reinforce the notion that *K. pneumoniae* infections acquired in community settings are more likely to involve susceptible strains, whereas those acquired during hospitalization are more likely to involve carbapenem-resistant strains as previously reported by (10, 17).

It is also important to highlight what factors did not significantly differ between the CRKP and CSKP groups in our analysis. Notably, gender was not a discriminator in the resistant vs. susceptible comparison, even though females predominated in overall CSKP infections. This suggests that, once infected with *K. pneumoniae*, the likelihood of that strain being carbapenem-resistant was more governed by age, setting, and comorbid conditions than by sex. Likewise, other comorbidities (renal disease, liver disease, malignancy) that differentiated infected patients from uninfected controls did not show a significant difference between CRKP and CSKP cases.

This may imply that those conditions elevate the risk of infection; however, among those infected, the evolution of resistance is more closely related to healthcare exposure and antibiotic pressure than to the presence of a particular underlying illness. Overall, our findings profile the typical CRKP patient as an older individual, often diabetic, who acquires the infection during a hospital stay – a profile consistent with global literature on carbapenem-resistant Enterobacteriaceae epidemiology (18).

The context of infection and resistance profile was another crucial determinant of outcome. The mortality model showed separate adjusted associations for hospital setting and carbapenem resistance; no interaction between these variables was tested. After adjustment for other covariates, individuals managed in hospitals had approximately 3.9-fold higher odds of death, while CRKP infection was associated with approximately 2.8-fold higher odds of death. These findings suggest that both healthcare-associated infection and CRKP may contribute to poorer clinical outcomes. This aligns with the consensus that infections caused by resistant strains have worse prognoses. Carbapenem resistance often implies delayed effective therapy and reflects a sicker inpatient population, thereby increasing mortality.

Prior studies have documented markedly higher mortality rates for CRKP infections, for example, roughly 40–42% mortality in CRKP bloodstream infections (BSIs) versus 20% for susceptible Klebsiella BSIs (19). Likewise, a report from China found in-hospital mortality of 42% for CRKP patients compared to 17% for CSKP (20). Our findings align with these, underscoring that resistance is a significant prognostic indicator. Moreover, hospital-acquired *K. pneumoniae* infections (which are often CRKP) carry additional risk due to the critical illness of patients and potential for multidrug-resistant co-infections.

In contrast, community-onset infections or those caused by susceptible strains are easier to treat and occur in relatively healthier hosts, resulting in better survival rates. The observed mortality differences are consistent with the independent associations of hospital setting and carbapenem resistance identified in the adjusted analysis. Because interaction between these variables was not formally tested, no inference regarding synergistic effects can be made. This reinforces the urgency of infection control and the need for appropriate empiric therapy for suspected CRKP in hospitalized patients.

Among underlying diseases, cardiovascular disease stood out as significantly associated with mortality. Patients with cardiovascular comorbidities had over three times the odds of death compared to those without (adjusted OR = 3.3, *p* = 0.002). This suggests that heart disease (such as coronary artery disease or heart failure) exacerbates the risk of dying from *K. pneumoniae* infection. Cardiovascular conditions can limit the physiologic reserve and perfusion during severe infection, and they often coexist with other risk factors like diabetes. Literature on bacteremia outcomes similarly lists chronic heart disease as a predictor of higher mortality (21–23).

Lastly, our mortality analysis uncovered significant relationship regarding the use of supportive devices. We found that patients who required any device support had higher odds of death compared to those who did not have devices (approximately 5.5 times greater mortality risk, *p* = 0.001). Similarly, previous studies have implicated invasive devices as risk factors for worse outcomes (21–23).

Our findings may reflect a specific nuance of our patient population or hospital practices. One possible interpretation is that some patients who died did so rapidly or had limitations of care, such that they never received escalated support (no time or no consent for intubation, for example), whereas survivors were more likely to have been aggressively supported with devices in the ICU. In essence, the absence of device use in the fatal cases might indicate that those patients were not stabilized enough or were not candidates for intensive intervention, leading to higher mortality.

Furthermore, explanation could be a survivor bias: patients managed with invasive devices may have initially been more critically ill, but the fact that they received advanced support might have improved their survival relative to those who never received such support. It is also worth noting that this counterintuitive association could be an artifact of our data or modeling.

Regardless, the result highlights that simply having intensive care interventions is not invariably a marker of poor outcome; in some cases, it may be a surrogate of receiving life-saving care. Our observation warrants cautious interpretation and suggests that outcomes in *K. pneumoniae* infection depend not only on illness severity but also on how and when intervention occurs. Future studies should explore this aspect; however, the overall message remains that the sickest patients (whether identified by need for ICU procedures or by underlying conditions) require prompt, aggressive management to reduce mortality. However, several adjusted estimates had wide confidence intervals, indicating limited precision and possible sparse-data bias. Therefore, these findings should be interpreted as exploratory rather than definitive and should be confirmed in larger multicenter cohorts.

This study has several strengths. A key strength of this study lies in its methodological design, which utilizes a robust case–case–control framework with explicit matching. By including both resistant and susceptible cases against a control group, the study provides a balanced and comprehensive comparative analysis. Furthermore, we have maintained a deliberately cautious and conservative approach to interpreting our findings, ensuring that our conclusions strictly reflect the data without overreaching. On the other hands, this study has a few limitations. Selection bias cannot be excluded because controls were selected retrospectively from patients with healthcare contact and no documented *K. pneumoniae* isolation. Culture practices may have differed according to clinical severity, ward location, and physician decision-making; therefore, some colonized or infected patients may have been misclassified as controls. Also, a major limitation is the absence of detailed antimicrobial exposure data, including prior carbapenem, cephalosporin, fluoroquinolone, and piperacillin-tazobactam exposure. ICU admission, severity of illness scores, prior hospitalization, invasive-procedure timing, and colonization status were also unavailable. These unmeasured variables may have confounded the observed associations, particularly those related to hospital setting, device use, and mortality. In addition, carbapenemase gene testing was available for only a subset of CRKP isolates; therefore, molecular resistance mechanisms could not be evaluated uniformly across the analytical cohort. Finally, because all isolates originated from urine specimens, findings may not be generalizable to bloodstream, respiratory, wound, or other invasive *K. pneumoniae* infections, and differentiation between urinary tract infection, asymptomatic bacteriuria, and urinary colonization could not be confirmed retrospectively for every patient. The retrospective design did not allow systematic application of standardized clinical infection criteria to every isolate. These findings are needed to be validated through larger multicenter studies.

## Conclusion

This study sheds light on distinct exploratory association patterns in *K. pneumoniae* infections and their outcomes. Female sex, non-Saudi nationality, and renal disease showed more stable adjusted associations with CSKP infection, whereas hepatic disease, malignancy, and absence of device use were imprecise model signals only. Older age and diabetes were associated with CRKP infection, while hospital exposure showed a directionally consistent but imprecise healthcare-associated signal. The mortality model showed associations with advanced age, hospital setting, CRKP infection, cardiovascular disease, and device utilization. The unexpected association between absence of device use and CSKP infection may reflect residual confounding or differences in infection source and should not be interpreted as evidence that device use is protective. These findings underscore the importance of targeted preventive strategies, such as rigorous infection control and Antimicrobial Stewardship program (ASP), to mitigate the impact of both susceptible and resistant *K. pneumoniae* infections. Furthermore, community studies are needed to assess population exposure to *K. pneumoniae*, and ASPs should be strengthened at PHCs alongside health-promotion campaigns regarding proper antimicrobial use for healthcare professionals and the public.

## Data Availability

The original contributions presented in the study are included in the article/supplementary material, further inquiries can be directed to the corresponding author.
